# 

**Published:** 1937

**Authors:** 


### CONTENTS.

*Page*

The Carey Coombs Memorial Lecture on the Pathology and Surgical Treatment of Cardiac Ischæmia. By LAURENCE O'SHAUGHNESSY, F.R.C.S. .. .. .. .. .. 109

Some Reflections on Research in Medicine. By F. J. POYNTON, M.D., F.R.C.P. .. .. .. .. .. .. .. .. .. 127

Spinal Anæsthesia. By A. W. ADAMS, F.R.C.S. .. .. .. 143

How Bristol Royal Infirmary has Watched the World Change. By E. WATSON-WILLIAMS, M.C., F.R.C.S. .. .. .. .. 167

Reviews of Books .. .. .. .. .. .. .. .. .. 171

Editorial Notes .. .. .. .. .. .. .. .. 180

Obituary: J. A. ARROWSMITH-BROWN, D.S.O. .. .. .. .. 183

Meetings of Societies .. .. .. .. .. .. .. 184

The Medical Library of the University of Bristol .. .. 185

Publications Received .. .. .. .. .. .. .. 187

Local Medical Notes .. .. .. .. .. .. .. 188

